# Differences in caecal microbiota composition and *Salmonella* carriage between experimentally infected inbred lines of chickens

**DOI:** 10.1186/s12711-022-00699-6

**Published:** 2022-01-29

**Authors:** Anaïs Cazals, Jordi Estellé, Nicolas Bruneau, Jean-Luc Coville, Pierrette Menanteau, Marie-Noëlle Rossignol, Deborah Jardet, Claudia Bevilacqua, Andrea Rau, Bertrand Bed’Hom, Philippe Velge, Fanny Calenge

**Affiliations:** 1 Université Paris-Saclay, INRAE, AgroParisTech, GABI, 78350 Jouy-en-Josas, France; 2 Mouse Genetics Laboratory, Department of Genomes and Genetics, Institut Pasteur, Paris, France; 3 Université François Rabelais de Tours, INRAE, UMR ISP, 37380 Nouzilly, France

## Abstract

**Background:**

*Salmonella* Enteritidis (SE) is one of the major causes of human foodborne intoxication resulting from consumption of contaminated poultry products. Genetic selection of animals that are more resistant to *Salmonella* carriage and modulation of the gut microbiota are two promising ways to decrease individual *Salmonella* carriage. The aims of this study were to identify the main genetic and microbial factors that control the level of *Salmonella* carriage in chickens (*Gallus gallus*) under controlled experimental conditions. Two-hundred and forty animals from the White Leghorn inbred lines N and 6_1_ were infected by SE at 7 days of age. After infection, animals were kept in isolators to reduce recontamination of birds by *Salmonella*. Caecal contents were sampled at 12 days post-infection and used for DNA extraction. Microbiota DNA was used to measure individual counts of SE by digital PCR and to determine the bacterial taxonomic composition, using a 16S rRNA gene high-throughput sequencing approach.

**Results:**

Our results confirmed that the N line is more resistant to *Salmonella* carriage than the 6_1_ line, and that intra-line variability is higher for the 6_1_ line. Furthermore, the 16S analysis showed strong significant differences in microbiota taxonomic composition between the two lines. Among the 617 operational taxonomic units (OTU) observed, more than 390 were differentially abundant between the two lines. Furthermore, within the 6_1_ line, we found a difference in the microbiota taxonomic composition between the high and low *Salmonella* carriers, with 39 differentially abundant OTU. Using metagenome functional prediction based on 16S data, several metabolic pathways that are potentially associated to microbiota taxonomic differences (e.g. short chain fatty acids pathways) were identified between high and low carriers.

**Conclusions:**

Overall, our findings demonstrate that the caecal microbiota composition differs between genetic lines of chickens. This could be one of the reasons why the investigated lines differed in *Salmonella* carriage levels under experimental infection conditions.

**Supplementary Information:**

The online version contains supplementary material available at 10.1186/s12711-022-00699-6.

## Background

Reducing *Salmonella* carriage in chicken flocks is important to ensure human food safety and enhance breeding viability. Indeed, consumption of contaminated raw poultry products can cause human food intoxications. Between 2014 and 2016, more than 4000 cases of human salmonellosis outbreaks due to the consumption of contaminated chicken meat and eggs were reported in Europe with more than 93% due to the Enteritidis serovar [1]. To decrease the number of cases, hygiene control measures, detection of serovars of *Salmonella*, and culling of contaminated flocks or vaccination have been implemented on farms. Nevertheless, the economic impact of human salmonellosis is estimated at approximately 3 billion euros per year [1]. Thus, developing new strategies to improve the control of *Salmonella* propagation in poultry livestock is essential to meet this challenge.

In adult chickens, *Salmonella enterica* Enteritidis (SE) does not induce symptoms and can remain in the host for a long time [2]. Carrier animals excrete the bacteria in the environment, thus increasing their propagation and facilitating contamination of other chickens. Previous studies have identified several quantitative trait loci (QTL) associated with *Salmonella* carriage in the White Leghorn inbred lines N and 6_1_ [3–5], which shows that differences in host genetics contribute to resistance to *Salmonella* carriage. However, the effects associated with these QTL are weak and do not allow direct application of marker-assisted selection for more resistant animals in commercial lines. The weak effects of these QTL might also result from biased quantification of *Salmonella* carriage in individual chickens, due to the recurrent recontamination of birds by *Salmonella* that are excreted by other birds within the timeframe of experimental infections. This recontamination probably leads to a homogenization of *Salmonella* carriage in chicken flocks. In fact, the use of isolators, which strongly reduces this recontamination, has led to much greater individual variation between birds in the experimental White Leghorn line PA12 [2], and has allowed the description of three categories of birds based on their level of shedding: super-, intermediate- and low-shedders. Through the use of grids on the floor and decontamination of faecal drops, isolators allow control of air purity and limit faecal-oral recontamination between birds.

In addition to the effect of host genetics, many studies have demonstrated the importance of the gut microbiota for the host’s health, not only in poultry, but also in other livestock species and in humans. In humans, disruptions of the intestinal microbiota can lead to many kinds of diseases by altering the host’s physiology and metabolism and triggering inflammation [6]. It is now clear that both the host and its intestinal microbiota contribute to the expression of many phenotypic traits of interest in livestock species [7]. In chickens, it is also well established that intestinal health is the result of complex functional interactions between intestinal microbes and host immunity [8]. In particular, the gut microbiota of adult chickens has been consistently identified as a protective factor to prevent colonisation of the intestine of young chicks by *Salmonella* sp. through a mechanism called competitive exclusion [9–11]. Although the primary site of *Salmonella* invasion in the host is the small intestine, the caecal microbiota plays a key role in susceptibility of the host to SE caecal colonization and on *Salmonella* shedding. The caecal microbiota composition before SE infection has been shown to be associated with the *Salmonella* super- or low-shedder phenotypes in the chicken lines N and 6_1_ [12]. And indeed, spraying adult microbiota onto young chicks or the use of probiotics are already effectively used in commercial flocks to reduce SE load [13–16]. Many studies have also assessed the effectiveness of nutritional strategies to reduce SE load by modulating the intestinal microbiota, which results in an improvement of the host immunity [17–20]. Such strategies include prebiotics or probiotics that boost production of beneficial metabolites, modulate host immunity, and improve function of the intestinal barrier [21].

To understand the underlying biological mechanisms explaining the reduction of SE load through nutritional strategies, and to identify the bacterial taxa realising the competitive exclusion of *Salmonella*, it is important to analyse the impact of *Salmonella* infection on host microbiota composition. By comparing infected and non-infected animals, several studies have identified Operational Taxonomic Units (OTU) signatures of *Salmonella* infection. For example in pigs, Argüello et al. [22] identified bacteria from the *Clostridia* class that could prevent *Salmonella* Typhimurium colonisation. In chicken, the *Ruminococcaceae* family, which is more abundant in non-infected animals at 4 days post-infection (dpi), could be a signature of *Salmonella* infection [23].

In conclusion, both host genetics and composition of the intestinal microbiota can explain differences in SE carriage in chickens. Host genetics and intestinal microbiota may also interact, since genetic studies of other phenotypes, such as digestive efficiency [24], body weight [25] or feather-pecking in laying hens [26], have shown that host genetics influences intestinal microbiota composition in chickens. In these studies, several QTL and single nucleotide polymorphisms (SNPs) that were associated with specific bacterial species and may explain the phenotype were identified, and significant heritabilities of the abundance of these bacteria were found.

In this study, we investigated the combined influence of host genetics and gut microbiota composition on caecal SE load. Using the N and 6_1_ inbred chicken lines, we studied the impact of the host genetics on both *Salmonella* carriage and microbiota composition at the individual level, using the isolator model, which highly decreases the exchange of gut microbiota between animals [2]. Our aims were to:

(i) Assess the individual variability in *Salmonella* load and in caecal microbiota composition within each line;
(ii) Compare *Salmonella* load and caecal microbiota composition of the two N and 6_1_ lines, to investigate the existence of a genetic control of these two phenotypes; and
(iii) Identify putative microbial signatures of low/high *Salmonella* carriage within and between the two lines.

## Methods

### Experimental design

**Fig. 1:**
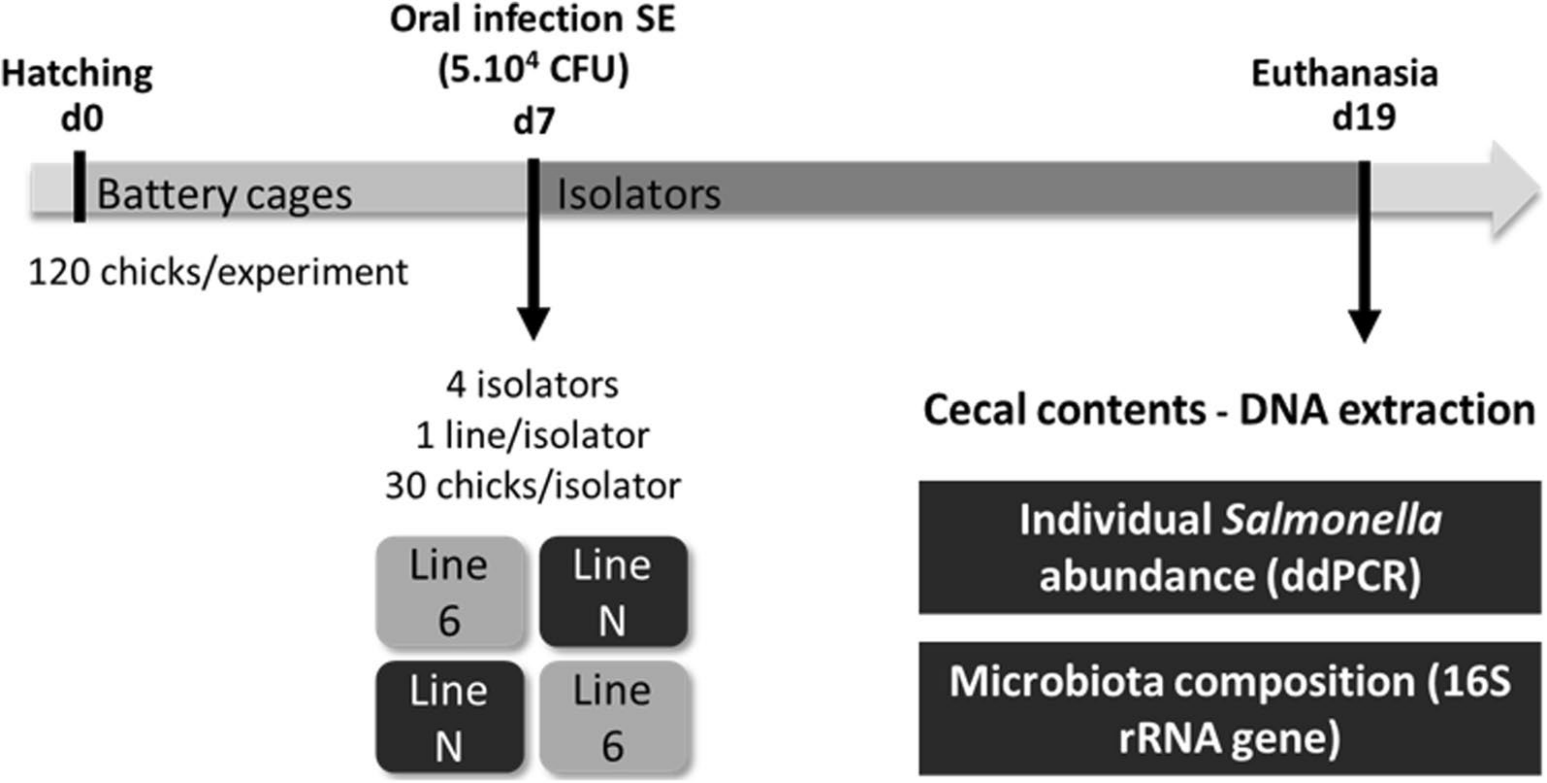
Experimental design. Two experiments were conducted, one in January 2017 and one in February 2017 for a total of 240 animals from the inbred White Leghorn lines N and 6_1_

Two experiments (i.e. Experiments 1 and 2) were conducted 1 month apart (January and February 2017) using birds from the White Leghorn inbred lines N and 6_1_. For each experiment (Fig. 1), chicks from the two lines (in total, n = 120; 30 males and 30 females from each line) were hatched together with free access to feed [for feed composition (see Additional file 1)] and water at the experimental unit PEAT (Pole d’Expérimentation Avicole de Tours, Nouzilly, France) and immediately transferred to the PFIE unit (Plateforme d’Infectiologie Expérimentale, INRAE, Nouzilly, France), where they were reared together on floor. At 7 days of age, each chick was orally infected with *Salmonella enterica* Enteritidis [Strain 775 (LA5 Nal20Sm500), 5 × 10^4^ cfu/0.2 mL/chick] and the chicks were transferred into four isolators to decrease oro-faecal recontaminations, as described previously [2], with two isolators for chicks from line N and two for chicks from line 6_1_, each isolator harbouring 30 birds. Sibs and half-sibs were separated between the two isolators for each line, in order to prevent confounding of a putative intra-line genetic effect on *Salmonella* carriage with the environmental effect of the isolator. Isolators with birds from line 6_1_ in Experiment 1 were used for birds from line N in Experiment 2 in order to prevent confounding of isolator effects with line effects. Then, at 12 dpi, animals were euthanized with CO_2_ according to the French regulation for experimental chickens. Caecal contents were gently collected to ensure that the intestinal mucosa was retained, weighed, transferred into cryotubes, and immediately frozen in liquid nitrogen and stored at − 80 °C until use. All animal procedures were approved by the Ethic committee (APAFIS#5833-2016062416362298v3) and authorised by the French Government.

### DNA extraction

Individual caecal DNA was extracted from ~ 200 mg of frozen caecal contents, as previously described [27]. Samples from Experiments 1 and 2 (see Fig. 1) were processed at the same time. In brief, samples were incubated at 70 °C for 1 h with 250 μL of guanidine thiocyanate buffer [4 M guanidine thiocyanate—0.1 M Tris (pH 7.5) and 40 μL of 10% *N*-lauroyl sarcosine—0.1 M phosphate buffer (pH 8.0)] and 500 μL of 5% *N*-lauroyl sarcosine. One volume (750 μL) of 0.1-mm-diameter silica beads (Sigma) was added and the tubes were shaken for 10 min at the maximum speed of a MM200 Mixer Mill (Retsch, Germany). Tubes were vortexed and centrifuged at 14,000 rpm for 5 min at 4 °C. After recovery of the supernatant, 30 μL of Proteinase K (Chemagic STAR DNA BTS kit, Perkin Elmer, USA) were added and samples were incubated for 10 min at 70 °C in a Multi-Therm shaker at 250 rpm (Benchmark Scientific, USA), then for 5 min at 95 °C for enzyme inactivation. The tubes were centrifuged at 14,000 rpm for 5 min at 4 °C and the supernatants were transferred into a deepwell plate. The plate was loaded onto the nucleic acid workstation Chemagic STAR (Hamilton, Perkin Elmer, USA) and DNA extraction was performed with the Chemagic STAR DNA BTS kit (Perkin Elmer, USA) by the @BRIDGe platform (INRAE, Jouy-en-Josas, France) according to the manufacturer’s instructions. DNA concentration was measured by fluorometric quantification (Qubit) and DNA samples were stored at − 20 °C.

### Quantification of *S.* Enteritidis by ddPCR

Individual abundances of SE in caecal contents at day 19 were obtained by Droplet Digital PCR using the QX200 Droplet Digital PCR system (Bio-Rad) on the @BRIDGe platform (INRAE, Jouy-en-Josas, France). Each DNA sample was diluted 1:2 and 1:5 in a final volume of 5 µL to be under PCR saturated conditions. Droplets were generated using samples of 20 µL taken from the amplification mix containing genomic DNA (15,000–20,000 droplets per sample). Amplification targeted a region of the *InvA* gene specific to SE using specific primers (forward: 5′-GCGTTCTGAACCTTTGGT-3′, reverse: 5′-CGTTCGGGCAATTCGTTA-3′) as described by Daum et al. [28] (see Additional file 1). PCR cycling conditions started with an enzyme activation step at 95 °C for 10 min, followed by 40 cycles at 94 °C for 30 min and 60 °C for 1 min, and ended by an enzyme deactivation step at 98 °C for 10 min. For all cycling steps, we used a 2.5 °C/s ramp rate.

For each sample, the number of copies of *Salmonella* per g of caecal content was calculated from the average number of copies of *Salmonella* per µL of the two dilutions, assuming that each amplified copy of the *InvA* gene corresponds to one *Salmonella* bacterium. Data were analysed with a log transformation of the copies of *Salmonella*. A Levene’s test was performed to test for differences in variation between groups. Analyses of variance (Anova) were performed to test the significance of differences in the number of copies of *Salmonella* between conditions (testing the effects “line”, “sex”, and “experiment”) using the Anova function from the car package (version 3.5-5) in the R software version 3.5.1 (Type II sum of squares), with a heteroscedasticity-corrected coefficient covariance matrix (white.adjust = TRUE) to account for differences in variances between groups.

### PCR and sequencing of the 16S rRNA genes

Samples from Experiments 1 and 2 were sequenced at the same time. Amplification of the V3-V4 hyper-variable region of the 16S rRNA coding gene was performed on the INRAE @BRIDGe platform. Universal V3-V4 primers (see Additional file 1) were used for the first PCR reaction. PCR cycling conditions were as follows: an initial denaturation step (94 °C for 10 min), 35 cycles of amplification (94 °C for 1 min, 68 °C for 1 min and 72 °C for 1 min) and a final elongation step at 72 °C for 10 min. Amplicons were then purified using magnetic beads (Clean NA, GC biotech B.V., The Netherlands). The DNA concentration was controlled using a Nanodrop spectrophotometer (Thermo Scientific, USA). In the second PCR, samples were multiplexed and another pair of primers was used (see Additional file 1) with the following PCR steps: an initial denaturation step (94 °C for 10 min), 12 cycles of amplification (94 °C for 1 min, 65 °C for 1 min and 72 °C for 1 min) and a final elongation step at 72 °C for 110 min. Amplicons were purified and the DNA concentration was controlled as described for the first PCR reaction. One run on an Illumina MiSeq was used to sequence amplicons (2 × 250 paired-end reads) according to the standard protocol.

### Bioinformatic and statistical analyses

Identification of OTU was performed using the FROGS pipeline [29]. The FastQC program was used to control quality and the Cutadapt program to find and remove adapter sequences from sequencing reads. R1 and R2 reads were merged and filtered (at Phred ≥ Q20) with the Flash program [30]. OTU were identified with the Swarm program [31]. Chimera OTU were removed by the VSEARCH program [32], and taxa filtering was performed with a minimum relative abundance threshold of 0.005% as proposed by [33]. Finally, phylogenetic affiliations were identified based on the Silva database by using the blastn + program [34]. OTU representing less than 0.5% of the global reads and samples with less than 10,000 reads were removed. For all the analyses involving diversity measures, rarefaction was performed using the rarefy_even_depth function in the phyloseq (1.24.2) package.

The phyloseq (1.24.2) and vegan (2.5-3) packages in R were used to perform diversity analyses on the rarefied data. Alpha diversity and beta diversity were measured based on the Shannon index and the Whittaker index, respectively. Anova were performed to test the significance of differences in alpha and beta diversities between conditions (line, sex, experiment, and isolator). Bray–Curtis dissimilarity was evaluated using the env_fit method by plotting in a non-metric multidimensional scaling representation. Permutational multivariate analyses of variance (Permanova) were used to test for significance of differences in diversity between lines, experiment, isolator, and sex.

The R package metagenomeSeq (1.24.1) was used on the unrarefied data to identify differentially abundant (DA) OTU between lines N and 6_1_ and between high and low carriers from line 6_1_. After normalisation of the OTU table, the model was fitted using the fitZIG method by including experiment, isolator, and sex as cofactors. Heatmaps were built on significant DA OTU using the function plotMRheatmap. Then, OTU were aggregated by family and genus with the command aggregateByTaxonomy to identify DA families and genera. Functional gene families and the MetaCyc pathway were predicted using the PICRUST2 package. DA KEGG orthologs (KO) and DA MetaCyc pathways were identified using the R package DESeq2 (1.26.0). DA MetaCyc pathways were aggregated at the super-pathway level based on the MetaCyc database [35].

## Results

### Abundance of *S.* Enteritidis in caecal contents

**Fig. 2:**
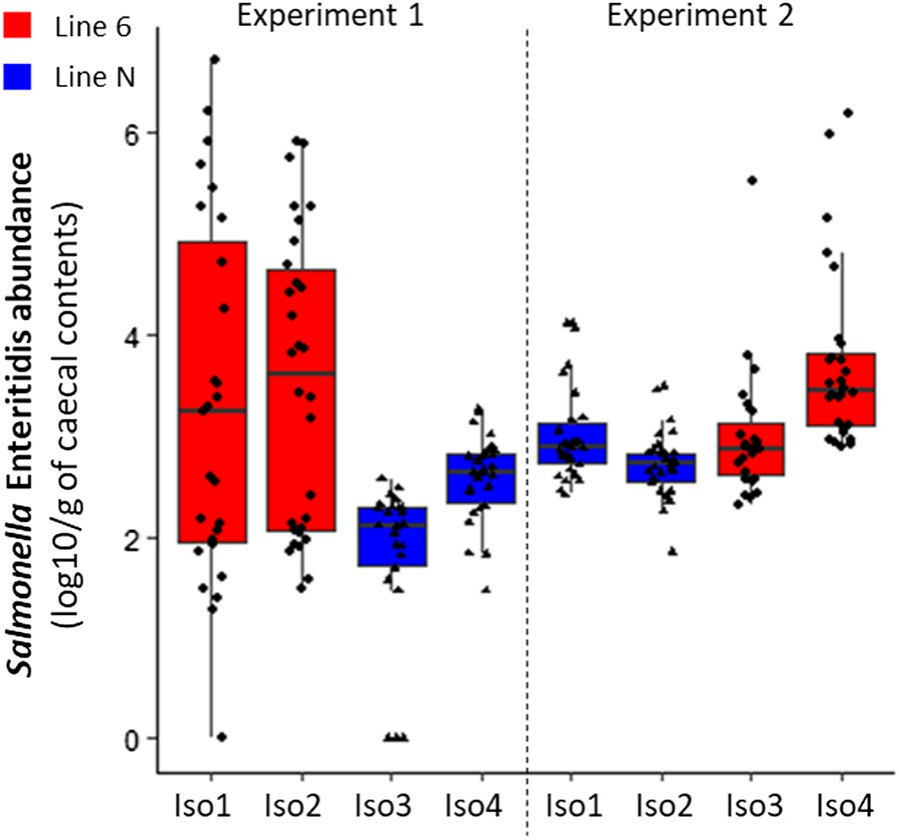
*Salmonella* Enteritidis abundance in caecal contents at 12 days post infection. *Salmonella* Enteritidis abundance at 12 days post infection in caecal contents of chickens from lines N and 6_1_ infected with *S*. Enteritidis (log 10/g of caecal contents) by experiment and isolator. *Salmonella* carriage is significantly different between lines and between experiments. In line 6_1_, the carriage is more variable between chickens, in particular for Experiment 1

After oral *Salmonella* infection at seven days of age, the chickens showed no clinical signs as expected with this dose of SE. The SE abundances in caecal content at 12 dpi from 228 chickens were well characterized by ddPCR, with an average of 3.38 and 2.55 log10 copies of DNA target sequences per g of caecal content for line 6_1_ and line N, respectively (Fig. 2). Since the Levene’s test showed differences in variance between groups (P < 0.001), we performed Anova using heteroscedasticity-corrected type II sum of squares by including line, experiment, and sex as factors, which revealed a significant difference (P < 0.001) in individual *Salmonella* carriage (ISC) between the two lines (Table 1) and (see Additional file 5: Table S1), which was also found when considering each experiment separately (Table 1). In addition, the ISC was found to be more variable in line 6_1_ than in line N (P < 0.001), with standard deviations of 1.3 and 0.7, respectively. The higher ISC variation in line 6_1_ was more marked in Experiment 1 than in Experiment 2 (P < 0.001), with standard deviations of 1.61 vs. 0.84 (Fig. 2). The average ISC did not differ significantly between males and females (see Additional file 5: Table S1). The average ISC was significantly different between the two experiments (P < 0.001) for line N (2.2 ± 0.7 in Experiment 1 vs. 2.9 ± 0.4 in Experiment 2) but not for line 6_1_. Differences between isolators were not significant for line 6_1_ in Experiment 1, but there were significant differences between isolators for line 6_1_ in Experiment 2 and between isolators for line N in Experiments 1 and 2 (P < 0.01).

**Table 1:** Mean and standard deviation of *Salmonella* Enteritidis abundance in caecal contents by line and experiment 12 days post infection and significance (P-value) of line effects based on Anova type II sum of squares test using all factors

Line	Experiment 1		Experiment 2		Both experiments	
	Number	Mean ± SD	Number	Mean ± SD	Number	Mean ± SD
N	56	2.21 ± 0.74	60	2.86 ± 0.43	116	2.55 ± 0.68
6_l_	57	3.41 ± 1.61	55	3.35 ± 0.84	112	3.38 ± 1.29
P-value		2.03e−06		2.12e−04		3.45e−09

In Experiment 1 for line 6_1_, hierarchical clustering identified two extreme groups of 15 chickens referred to as low and high carriers and for which usable information on microbiota was available. The mean abundance was 1.8 ± 0.6 and 5.2 ± 0.6 log10 copies of DNA per g of caecal content for the low and high carrier groups, respectively.

### Structure and diversity of the bacterial communities

Metadata, unrarefied OTU and the corresponding taxonomic classifications are in Additional files 2, 3 and 4, respectively. Due to strict controls on DNA quality after extraction and amplification, samples with at least 10 ng of DNA from 228 of the 240 chickens were sequenced. Sequencing resulted in 8,300,144 reads, with on average 32,464 reads per sample. From these reads, 617 OTU were identified, with percentages of affiliation of 99 and 65% at the family and genus levels, respectively. Samples with less than 10,000 total reads were removed, leaving data on 182 chickens, 86 and 95 from lines 6_1_ and N, respectively. The excluded samples were distributed across lines, genders, experiments, and isolators.

**Fig. 3:**
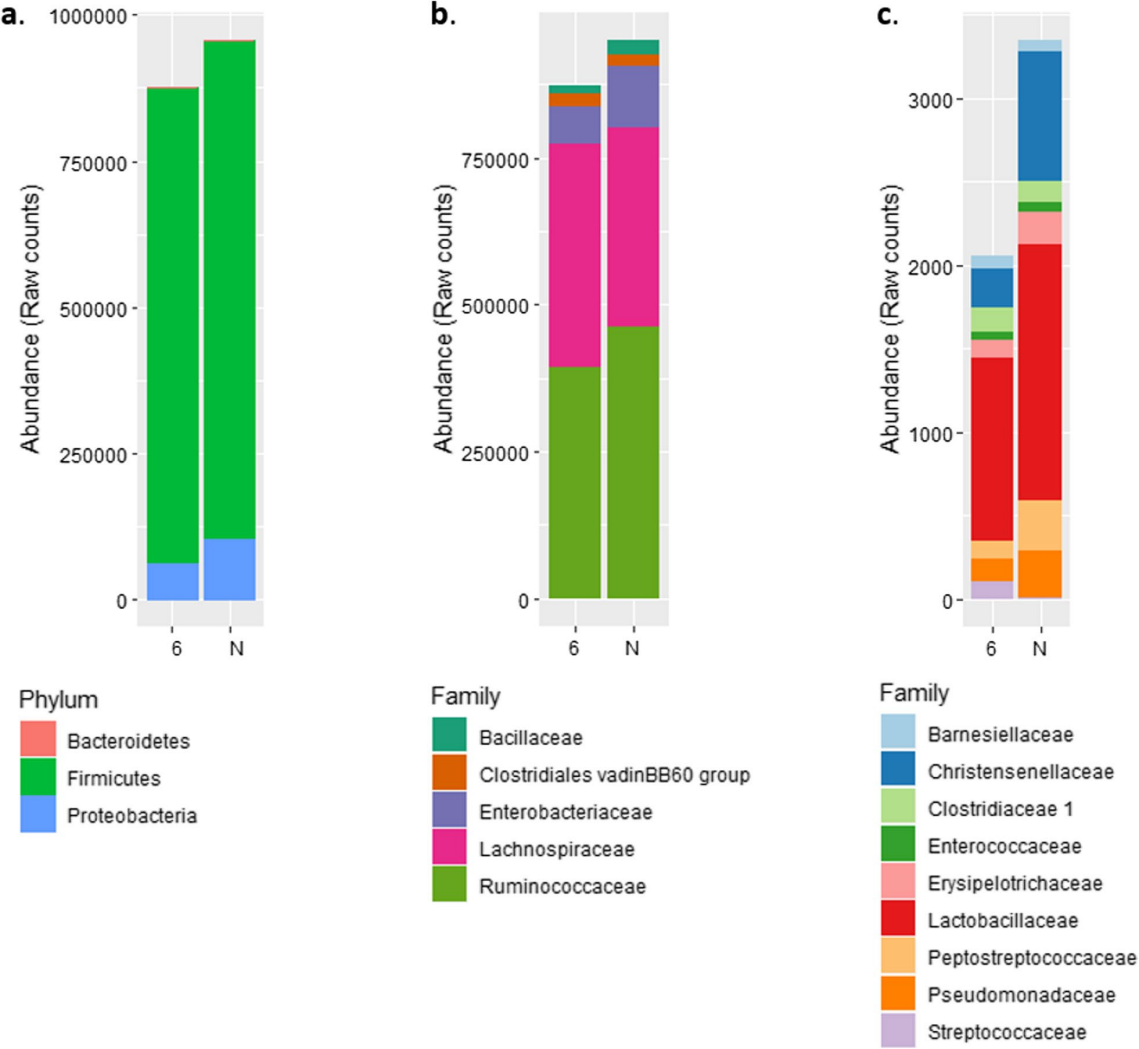
Microbiota composition at phylum and family levels for lines N and 6_1_. Microbiota composition for lines N and 6_1_ using raw counts after rarefaction at the phylum level (**a**), at the family level for the most abundant taxa (**b**) and at the family level for the least abundant taxa (**c**). The most abundant phylum is *Firmicutes* followed by *Proteobacteria* and *Bacteroidetes*. There is no significant difference at the phylum level between lines. At the family level we observed, with decreasing abundances, *Ruminococcaceae* and *Lactobacillaceae*, followed by *Enterobacteriaceae*, *Clostridiales* and *Bacillaceae*

At the phylum level, *Firmicutes* dominated the bacterial composition, followed by *Proteobacteria* and *Bacteroidetes* (Fig. 3a). At the family level, we observed a predominance of *Lachnospiraceae* and *Ruminococcaceae*, followed by *Enterobacteriaceae*, *Clostridiales* and *Bacillaceae* (Fig. 3b) and (see Additional file 5: Table S2).

**Fig. 4:**
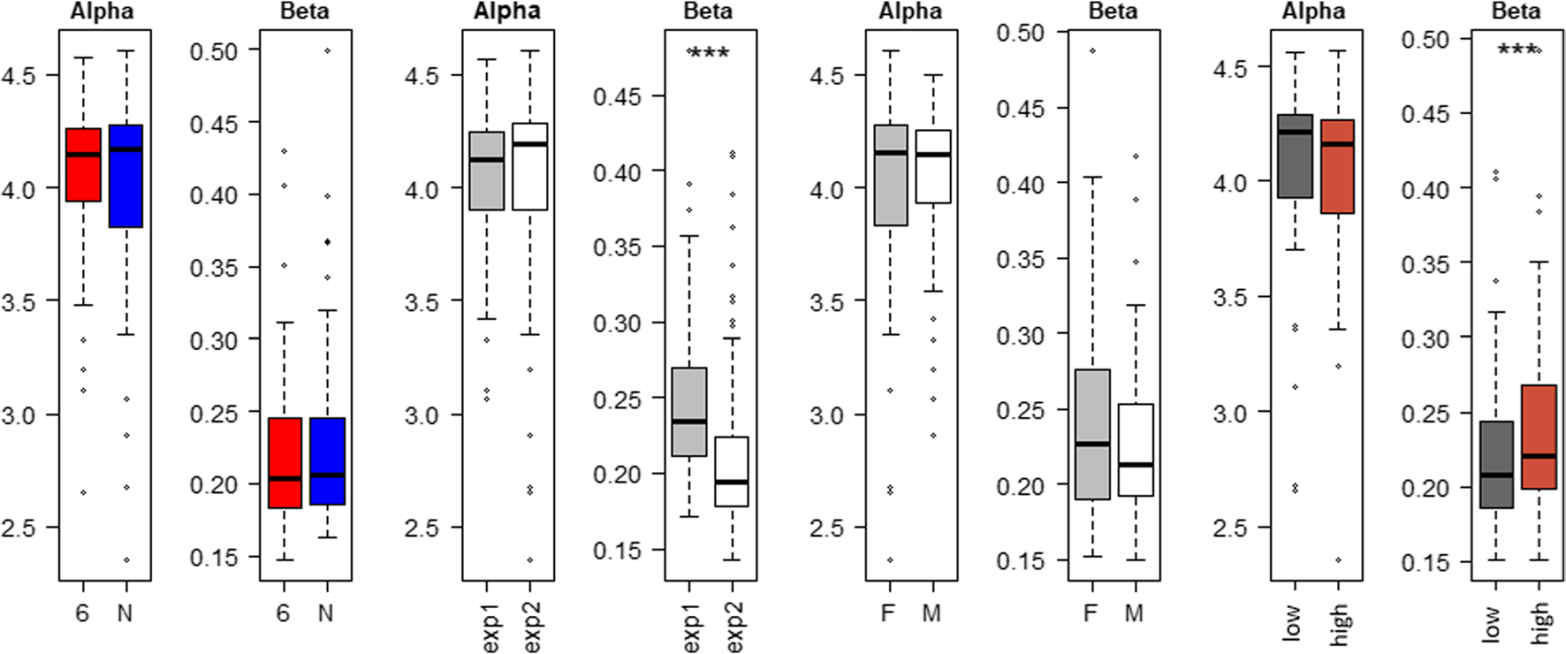
Alpha- and beta-diversity between lines, experiments, sexes, and between high and low carriers from line 6_1_. Using the Shannon index, alpha diversity is not significantly different between lines, experiments, sexes, and between high and low carriers from line 6_1_. Using the Whittaker index, beta diversity is significantly different between experiments (P < 0.0001) and between high and low carriers from line 6_1_ (P < 0.01), but not between lines and sexes

Analysis of the alpha diversity with the Shannon index did not show significant differences between lines, experiments, or sexes (Fig. 4) and (see Additional file 5: Table S3). Using the Whittaker index, we observed a significant difference in beta diversity between experiments (P < 0.0001), with 0.24 for Experiment 1 vs. 0.20 for Experiment 2. However, we did not observe a difference in beta diversity between lines (0.20 for both lines). High- and low-carriers from line 61 did not significantly differ in alpha diversity and richness but did in beta diversity (Fig. 4) and (see Additional file 5: Table S3).

### Non-metric multidimensional scaling and differentially abundant OTU between lines

**Fig. 5:**
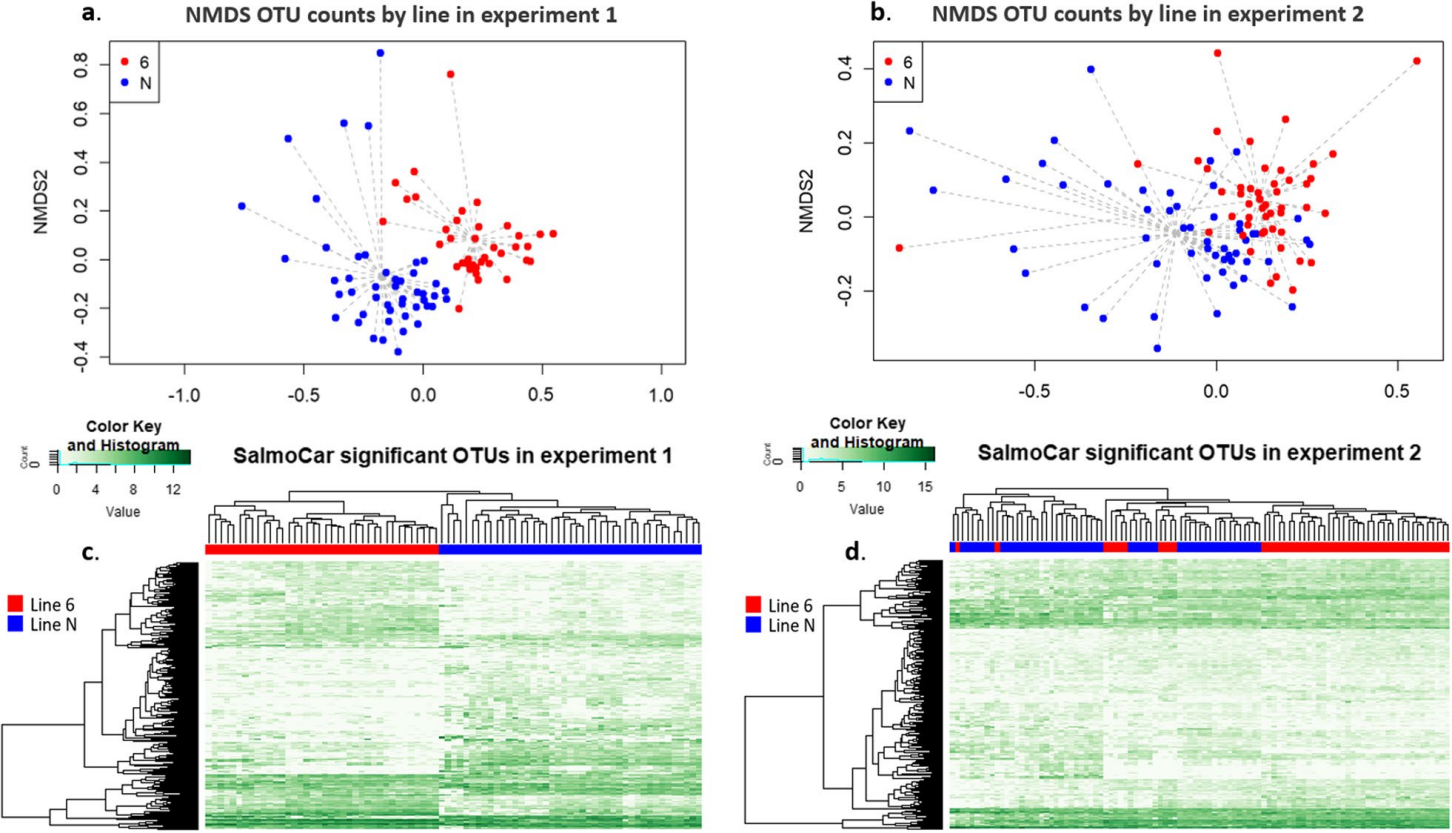
Non-metric multidimensional scaling representations and heatmaps comparing caecal microbiota composition between lines N and 6_1_. Non-metric multidimensional scaling representations of the OTU composition of caecal contents using the Bray–Curtis dissimilarity. Comparison of composition between lines N and 6_1_ in Experiment 1 (**a**) and Experiment 2 (**b**). A significant difference in microbiota composition is observed between lines after correction for isolator and sex effects (Permanova, P < 0.001). Heatmaps of the caecal OTU with differential abundances between lines N (in blue) and 6_1_ (in red) in Experiment 1 (**c**) and in Experiment 2 (**d**)

Analysis of the beta diversity using Bray–Curtis dissimilarity and an NMDS representation (Fig. 5) showed that the microbiota composition clearly clustered by line. This clustering was observed for both experiments but in particular for Experiment 1 (Fig. 5). The Permanova analysis confirmed this line effect (P < 0.001) after correcting for experiment, isolator, and sex effects, regardless of whether the data from Experiments 1 and 2 were merged or analysed separately (see Additional file 5: Table S4). The microbiota composition also differed significantly between the two experiments and between isolators but not between males and females (Additional file 5: Table S4).

Among the 617 identified OTU, 390 were significantly differentially abundant (DA) between lines across the two experiments, after adjusting for isolator effects (see Additional file 5: Table S5). When the two experiments were analysed separately, the heatmap showed a more strongly defined clustering for Experiment 1, with 388 DA OTU between lines (Fig. 5), than for Experiment 2, with 284 DA OTU. In total, 187 common DA OTU were found between the two experiments.

**Fig. 6:**
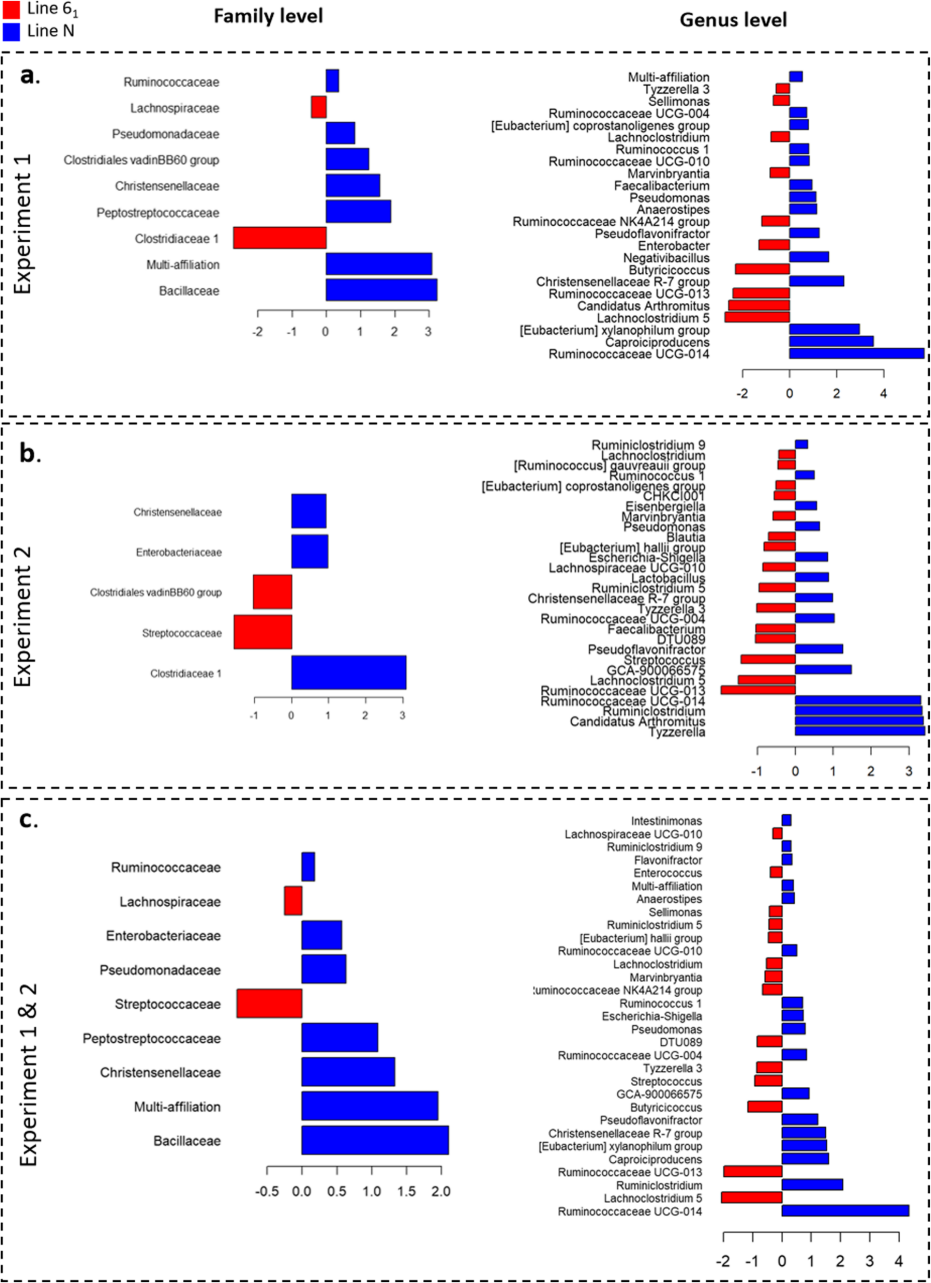
Differentially abundant families and genera between lines N and 6_1_ in Experiments 1 and 2. Differentially abundant families and genera between lines N and 6_1_ for Experiment 1 (**a**), Experiment 2 (**b**), and Experiments 1 and 2 together (**c**). Representation of the log_2_ fold-changes for the comparison between lines (blue indicates greater abundance in line N, red greater abundance in line 6_1_)

The 617 OTU were aggregated in order to compare compositions at higher taxonomic ranks, which led to 14 families and 51 genera. Nine DA families and 31 DA genera were identified between the two lines over the two experiments, after adjusting for isolator effects (Fig. 6) and (see Additional file 5: Table S6). For Experiments 1 and 2, nine and five DA families and 24 and 29 DA genera were identified, respectively (Fig. 6). At these levels of aggregation, families and genera that were significantly DA between the two lines were not the same for the two experiments, and some were inversely abundant. Nevertheless, in both experiments, the *Christensenellaceae* family was more abundant in the resistant line N. At the genus level, *Tyzzerella 3*, *Lachnoclostridium*, *Marvinbryantia*, *Ruminococcaceae UCG-013* were more abundant in line 6_1_ (susceptible) and *Ruminococaceae UGC-004*, *Ruminococcus 1*, *Pseudomonas*, *Pseudoflavonifractor*, *Christensenellaceae R-7 group* and *Ruminococcaceae UGC-014* were more abundant in line N (resistant).

### High and low carriers from line 6_1_ and the family *Christensenellaceae*

**Fig. 7:**
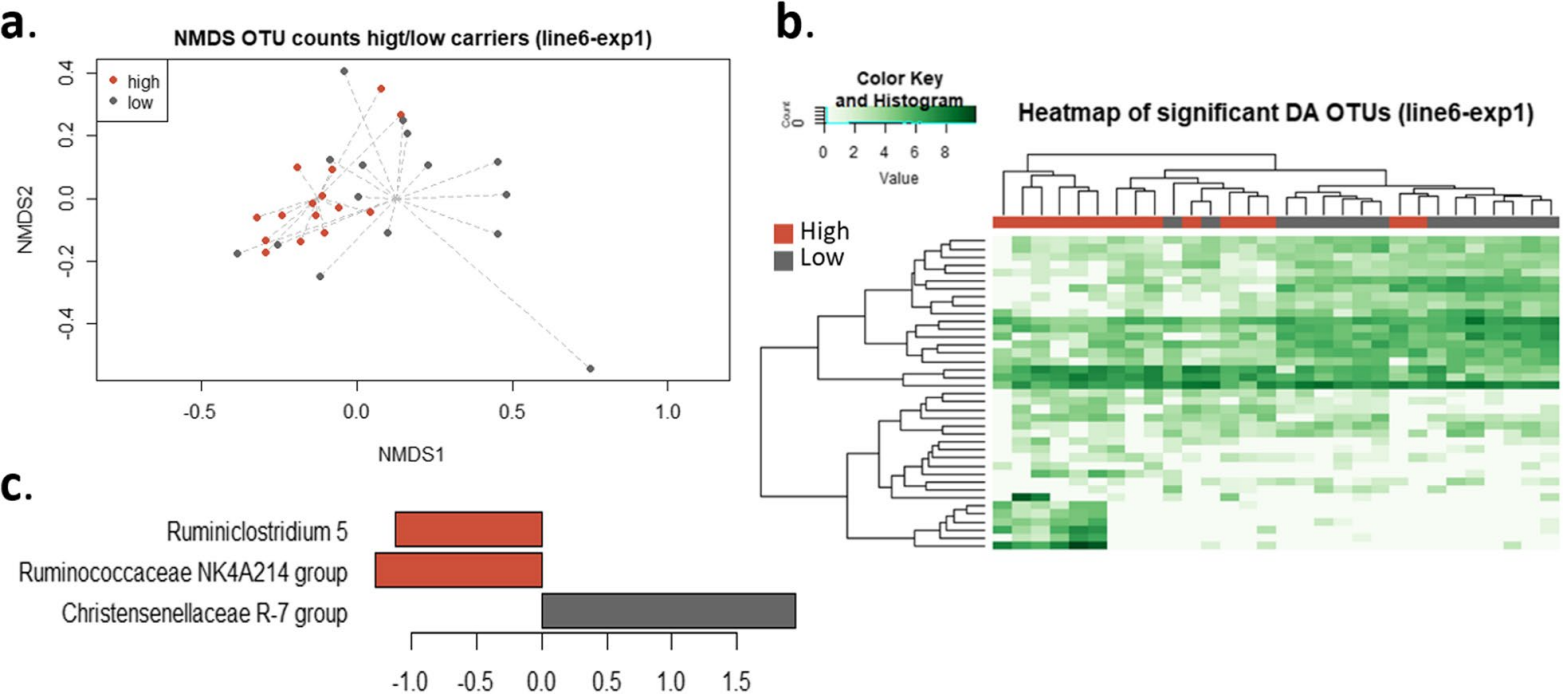
Non-metric multidimensional scaling representation, heatmap, and significant differentially abundant genera for high and low carriers (line 6_1_- exp1). **a** Non-metric multidimensional scaling representation of the caecal microbiota of high and low carriers from line 6_1_ (Bray–Curtis dissimilarity). High and low carriers are significantly different (Permanova, P-value < 0.01); caecal microbiota from different isolators are not significantly different (Permanova, P-value > 0.5). **b** Heatmap representation of the 39 differentially abundant OTU between high- and low-carriers from line 6_1_ in Experiment 1. **c** Differentially abundant genera between high- and low-carriers from line 6_1_ in Experiment 1 (Log_2_ fold-changes)

An NMDS representation for Experiment 1 was used to assess a potential difference in microbiota composition between high and low carriers from line 6_1_ (Fig. 7a). Experiment 1 was used for this analysis because it had the largest variability in individual *Salmonella* carriage, which allowed for the identification of high and low carriers. In Experiment 1, the Permanova analysis showed a significant (P < 0.01) difference in microbiota composition between the high and low carriers. DA analysis using the metagenomeSeq package identified 39 DA OTU between the high and low carriers (Fig. 7b) and (see Additional file 5: Table S7). After aggregation, one DA family (*Christensenellaceae*) and three DA genera (*Ruminococcaceae NK4A214*, *Ruminiclostridium 5*, and *Christensenellaceae R-7* groups) were identified (Fig. 7c) and (see Additional file 5: Table S8).

A comparison of results from the DA analyses between lines, on the one hand, and between high and low carriers, on the other hand, led to the identification of one common DA family, i.e. *Christensenellaceae*, which was more abundant in low carriers than in high carriers from line 6_1_ and was also more abundant in chickens from line N, which are more resistant (i.e. low carrier) to *Salmonella.* The same was observed for the DA genus *Christensenellaceae R-7 group*, which was more abundant in low carriers and in the resistant line N in both experiments. Regression analysis identified a significant correlation (P-value = 4.11e^−06^) between ISC and *Christensenellaceae* abundance.

### Functional gene prediction

**Fig. 8:**
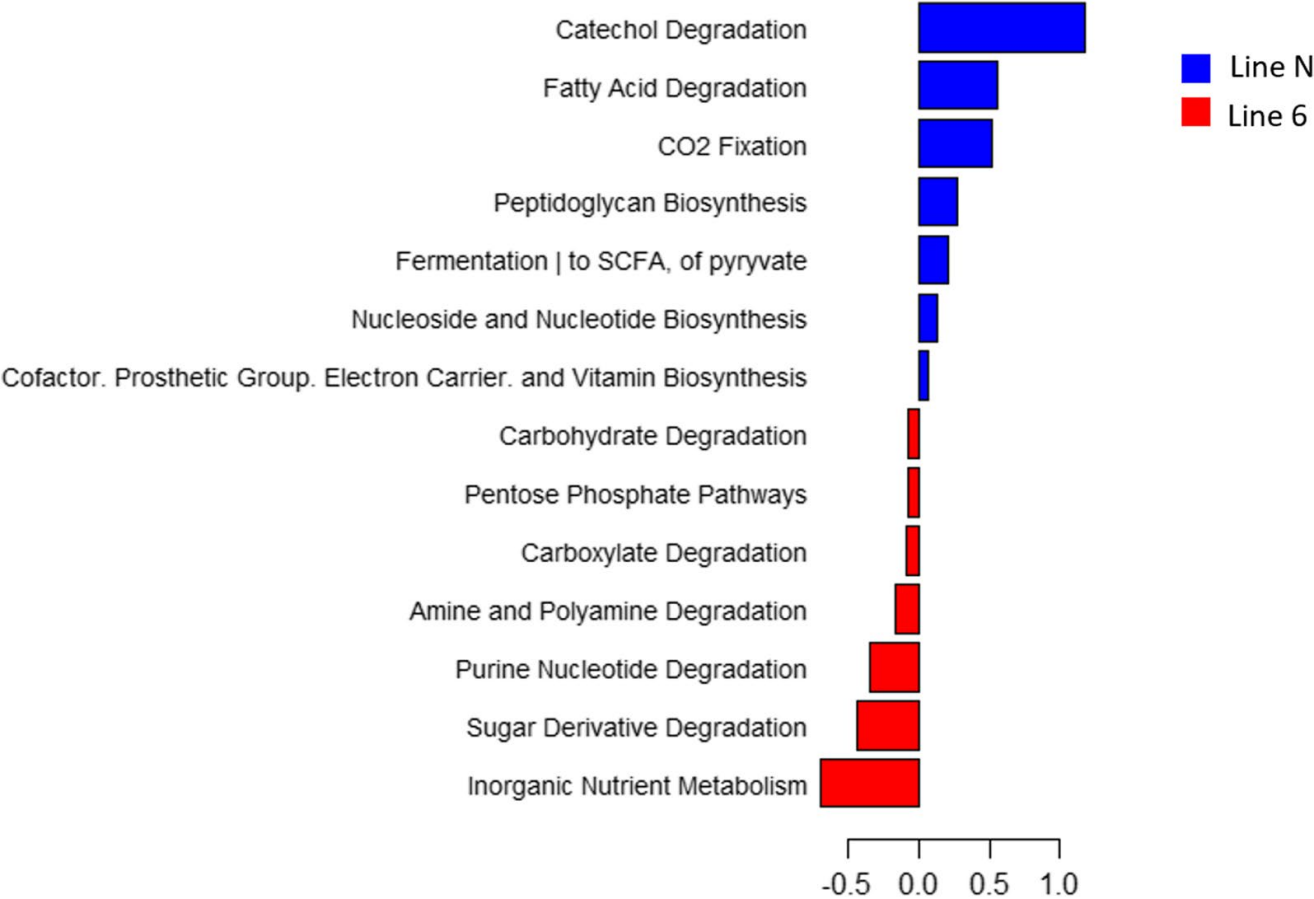
Common differentially abundant super-pathways between lines N and 6_1_ and between high and low-carriers from line 6_1_. Common differentially abundant super-pathway (MetaCyc database—DESeq2) in the comparison of caecal microbiota between lines N and 6_1_ and between high and low carriers from line 6_1_. Representation of the log_2_ fold-changes for the comparison between lines (blue indicates greater abundance in line N and red greater abundance in line 6_1_)

By using the PICRUST2 software, 4954 KEGG orthologs (KO) and 323 pathways were predicted based on the OTU table. The DESeq2 package identified 1590 DA KO and 69 DA pathways between lines N and 6_1_, after adjustment for the effect of experiment, (false discovery rate (FDR) < 0.01). For line 6_1_, the analysis between high and low carriers in Experiment 1, after adjustment for isolator effects, resulted in 507 DA KO and 75 DA pathways (FDR > 0.01). The 64 common DA pathways between lines N and 6_1_ and between high and low carriers from line 6_1_ were aggregated at the super-pathway level (Fig. 8) and (see Additional file 5: Table S9).

## Discussion

Using a model of *Salmonella* infection on chickens placed in isolators, which limits animal to animal recontaminations and animal reinfection and, thus, homogenisation of individual *Salmonella* carriage [2], we confirmed that individual *Salmonella* carriage varied both with the host genetics and microbiota composition. We identified differences in gut microbiota that were associated both with genetic line (N vs. 6_1_) and the individual *Salmonella* carriage status (high vs. low ISC carriers). Finally, the caecal microbiota taxonomic analysis identified taxa and metabolism pathways that may be associated with *Salmonella* carriage.

In both Experiments 1 and 2, line N was more resistant than line 6_1_ (based on the ISC phenotype), and the microbiota taxonomic composition also differed significantly between the two lines. Nevertheless, ISC was higher in Experiment 2, especially for line N, and the OTU that were DA between lines were not entirely the same between Experiments 1 and 2. Differences in microbiota composition between the two experiments might be due to the differences in environment. Although conditions were strictly controlled to be identical between the two experiments, slight differences in the environment may occur, resulting in different microbial compositions in the environment, which affects the primo-colonisation of the gut by bacteria in new-born chicks. Two studies have reported differences in the primo-colonisation of the intestinal tract between chickens, even when they are reared together in the same environment [36, 37]. Differences in microbiota composition before infection could affect microbiota composition after infection. Because of differences in ISC and microbiota composition between the two experiments, we will focus only on the results that were validated in both experiments in the following section.

### Impact of the chicken line on individual *Salmonella* Enteritidis carriage and microbiota composition

Using different infection models, previous studies have shown the impact of host genetics on *Salmonella* carriage and significant differences in SE carriage between commercial or local chicken breeds [38–40]. Several candidate genes have been shown to be associated with resistance to SE carriage, such as *SLC11A1* and *TLR4* [41–43]. Our results with lines N and 6_1_ confirm previously reported results for the same two lines without using isolators and with on-floor grouped rearing [3, 44], which confirm that line N is a lower SE carrier than line 6_1_. In addition, several QTL have been identified in different populations that derive from N × 6_1_ crosses [3–5]. Interestingly, our two independent experiments confirmed a larger variability in ISC for line 6_1_ than for line N, which raises new hypotheses, i.e. whether this difference in variability in ISC is caused by genetic differences between the lines, or by a differences in microbiota composition.

In the two experiments conducted here, the significant difference in caecal microbiota composition between lines N and 6_1_ at 12 dpi suggests the presence of host genetic control of this composition. For each experiment, birds from the two lines were hatched in the same environment and at the same time, and raised together until the experimental infection, such that the initial microbial environment was similar for all birds tested before infection. Thus, differences in caecal microbiota composition between lines cannot be attributed to differential exposures to environmental microbes before infection. Furthermore, for Experiment 1, we observed no significant difference in microbiota composition between the two isolators that were used for a given line [P > 0.1 (see Additional file 5: Table S4)]. Thus, in Experiment 1 we cannot associate differences in microbiota composition between the two lines with an effect of the isolator environment. In contrast, in Experiment 2, a significant difference in microbiota composition was observed between the two isolators used for a given line [P < 0.01 (see Additional file 5: Table S4)]. One can argue that the isolators were populated by different microbial populations, which could in turn influence the caecal microbiota composition after infection, but in this study the isolators were sterilized before each experiment. In addition, although the isolators used for each line were switched between the two experiments, observed differences in caecal microbiota composition between the two lines were similar for the two experiments. Moreover, since the isolators decrease oro-faecal recontamination of commensal microbiota and of pathogenic bacteria, the caecal microbiota composition of each chicken matured in isolation from the impact of the other birds once the animal has been put into the isolator.

Since we analysed caecal microbiota composition after infection, we cannot exclude that it was affected by the infection with *Salmonella*. Does the higher susceptibility of line 6_1_, with a larger number of *Salmonella* in the caeca, cause a shift in microbiota composition? Several studies have shown that SE infection has a very low impact on the composition of the caecal microbiota [23, 45–47], even if some differences in taxa abundance have been identified between infected and uninfected chicken [47, 48]. Thus, we hypothesize that, in our study also, SE infection had a weak impact on microbiota composition.

In a study of *Campylobacter* carriage between lines N and 6_1_, microbiota were transferred from the resistant to the susceptible line [49]. Although microbiota composition did not differ between the donor birds from the N and 6_1_ lines at 21 days of age, the genetic line of the recipient had a significant impact on microbiota composition in the recipient after transplantation, while the donor line of the transplanted microbiota did not. This supports our hypothesis that the host genetics has a strong impact on the composition of the microbiota in lines N and 6_1_ at 3 weeks after hatching and that genetic differences between lines N and 6_1_ might indirectly influence *Salmonella* carriage by impacting the microbiota composition. However, this does not exclude other potential pathways, in particular those involving host immunity.

In chickens, as well as in other livestock species and in humans, host genetic control of intestinal microbiota composition is well documented. In studies on humans, the abundance of some bacteria that are known to be associated with host immunity is heritable and host candidate genes have been identified [50]. In chickens, some studies have identified differences in microbiota composition between genetic lines, for instance between two lines that differ in their susceptibility to bacterial infections [51], between two divergent lines for body weight [52], and between four commercial lines and an indigenous Indian breed [53]. At least two studies have reported moderate estimates of heritability for bacteria family abundances and have detected several QTL involved in the control of these bacterial abundances by the host [24, 54].

### Relationships of caecal microbiota with individual *Salmonella* Enteritidis carriage

Colonisation and adhesion of commensal bacteria on the mucosal epithelium constitute a protective biofilm due to their competitive exclusion (CE) function. Studies affirm that CE is currently the best approach to decrease *Salmonella* colonization in commercial production of chickens [10]. In our study, all animals were experimentally infected and signature bacteria were identified by comparing chickens with differences in individual *Salmonella* carriage between or within lines. Our hypothesis is that the abundance of commensal, potentially competitive bacteria, is higher in the microbiota of resistant chickens, thus preventing colonisation of *Salmonella* in the intestinal tract. Host genetics could, in part, control this abundance of competitive bacteria.

From our results, it appears that the difference in ISC between lines and the larger variability of ISC in line 6_1_ cannot be related to a difference in beta- or alpha-diversity of the caecal microbiota between the two lines. Other studies that compared chickens from different breeds or lines found no significant differences in the Shannon index between birds that were infected with SE versus those that were not at 14 dpi [47, 55] or at 10 dpi [23]. These observations corroborate our findings and lead us to the conclusion that neither infection with SE nor the level of SE carriage affect OTU diversity in the caecal microbiota. However, we identified a significant difference in beta diversity between high and low carriers from line 6_1_, in that microbiota of high carriers were more similar to each other than those of low carriers. This raises the question whether the higher level of *Salmonella* drives the microbiota to a more similar composition. Or are high carriers more susceptible to *Salmonella* because some shared characteristics of their microbiota lead to a less efficient competitive exclusion? These questions remain open, as inbred lines N and 6_1_ are not fully homozygous, so that residual genetic variations within line 6_1_ may explain these differences in caecal microbiota beta diversity.

Even in the absence of differences in alpha- or beta-diversity, the DA OTU that were identified between the two lines could be associated with differences in ISC (Fig. 6). Likewise, the DA OTU that were identified between high and low carriers from line 6_1_ could be associated with ISC (Fig. 7).

We observed that the *Christensenellaceae* family was more abundant in low *Salmonella* carriers (Figs. 6 and 7). This bacteria family is associated with a beneficial impact on health in humans and in mice [56–59]. Interestingly, it has been shown that *Christensenellaceae* is one of the most heritable bacterial families of the human intestinal microbiota [56]. This leads us to formulate the hypothesis that host genetic differences between lines N and 6_1_ cause differences in *Christensenellaceae* abundance, which in turn could affect *Salmonella* resistance. However, to our knowledge, the heritability of the abundance of *Christensenellaceae* has not been assessed in chickens. Similarly, the family *Enterobacteriaceae* was more abundant in the resistant line (Fig. 6). These bacteria are in competition with *Salmonella* for oxygen [60] and for the use of nutrients such as iron [61] and are also able to produce bacteriocin that inhibits the proliferation of *Salmonella* [60]. Oxygen respiration by competitive bacteria such as *Enterobacteriaceae*, leads to anaerobisation of the lumen and, thus, a decrease in the capacity of *Salmonella* colonisation [60]. Our results are compatible with this hypothesis. Finally, the *Ruminococcaceae* family was less abundant in the susceptible line 6_1_ (Fig. 6). It has been shown that a decrease in *Ruminococcaceae* can be associated with an increase in inflammation [62] and thus an increase in *Salmonella* competition [63].

Based on the imputed putative functions associated with the DA OTU, based on known databases, some of these DA OTU harboured striking functions. Interestingly, several of the potential functions associated with the DA OTU may explain the observed differences in ISC between lines and between high and low carriers within line 6_1_. Although very speculative since it is based on an indirect, in silico approach, the significant functions identified are highly consistent with known mechanisms of resistance to *Salmonella*. First, the short-chain fatty acids (SCFA) metabolic pathway may be more abundant in both the resistant line N and in low carriers from the susceptible line 6_1_. Consequently, the production of SCFA could be associated with low *Salmonella* carriage. Many studies have shown that the production of SCFA by the intestinal microbiota, such as butyrate, has beneficial effects for the host [64–66]. In chickens, butyric acid can decrease inflammation [67], which is unfavourable for *Salmonella* [63]. More specifically, it has been shown that increasing SCFA in in vitro cultures of avian intestinal cells decreases the pathogenicity of *Salmonella* by blocking its entry into the host [68]. Thus, line N and the low carriers from line 6_1_ could carry a beneficial microbiota for *Salmonella* resistance. Second, the catechol degradation pathway may be more abundant in both the resistant line N and in the low *Salmonella* carriers from the susceptible line 6_1_. *Salmonella* have the ability to produce auto-inducers 3 (AI-3), which have a similar chemical structure as the catecholamine from the catechol family [69, 70]. In chickens and pigs, treatment with a catecholamine such as norepinephrine, is known to increase *Salmonella enterica* colonization in the host and *Salmonella* spread in the environment [71, 72]. Catechol also plays a role in quorum sensing signalling for the production of biofilm, thus increasing the virulence of *Salmonella* during host infection [69, 73] and the capacity of *Salmonella* to persist on eggs or meat [74]. Thus, the capacity of microbiota for catechol degradation in line N and in the low carriers from line 6_1_ could be beneficial for *Salmonella* resistance.

Nevertheless, bacteria that are potentially beneficial to health have also been found with a higher abundance in the susceptible line. For example, the *Blautia* genus, which is more abundant in line 6_1_ in Experiment 2 (not confirmed in Experiment 1), is a butyrate producer [75] and has beneficial anti-inflammatory effects [76]. Thus, looking at individual taxa might not be sufficient, and we suggest that the total balance of beneficial bacteria has an impact on the *Salmonella* resistance, which supports the relevance of studying the aggregated contributions of several taxa to the same metabolic pathway.

## Conclusions

Our findings show that ISC in caeca and caecal microbiota taxonomic composition differ between the N and 6_1_ inbred lines of chickens. The results also show associations of ISC status (high vs. low carriers) with the abundance of bacterial taxa and metabolic pathways that were previously associated with resistance to SE. Most notably, we identified an overrepresentation of the short chain fatty acids metabolic pathway and the catechol degradation pathway in low carriers, as well as of the *Christensenellaceae, Enterobacteriaceae* and *Ruminococcaceae* families. Based on these observations, we hypothesize that genetic differences between lines N and 6_1_ may influence the level of *Salmonella* carriage by influencing the abundance of beneficial bacteria. Combining information on host genetics and gut microbiota composition is useful to increase the accuracy of prediction of complex traits such as resistance to pathogens. Our study showed that both caecal microbiota and host genetic background play a role in mechanisms that lead to *Salmonella* colonization resistance in chickens. Future studies should decipher the host genes that control differences in bacterial abundances in these model lines and study their effects in other genetic backgrounds and environments.

## Supplementary Information

**Additional file 1. **Feed composition used, primers sequences used for the amplification of the region specific to *Salmonella* Enteritidis in the *invA* gene and adapter/primer used in Illumina amplicon library preparation.

**Additional file 2. **Metadata associated with all samples.

**Additional file 3. **Unrarefied operational taxonomic unit table.

**Additional file 4**. Taxonomic assignments for operational taxonomic units.

**Additional file 5: Table S1.** Results of the Anova test on individual Salmonella carriage for line, sex and experiment. **Table S2.** Proportion of phylum and family types over the 228 caecal microbiota analysed. **Table S3.** Results of the Anova test on alpha diversity and beta diversity for line, sex, experiment and high- and low-carriers. **Table S4.** Results of the Permanova on Bray–Curtis distance for experiment, isolators, sex and line. **Table S5.** Differentially abundant operational taxonomic units (DA OTU) between the N and 6_l_ lines over the two experiments, when adjusting for isolator effect. **Table S6.** Differentially abundant genera and families between the N and 6_l_ lines over the two experiments, when adjusting for isolator effect. **Table S7.** Differentially abundant operational taxonomic units (DA OTU) between the high and low carriers (line 6_l_-Experiment 1), when adjusting for isolator effect. **Table S8.** Differentially abundant genera and families between high and low carriers (line 6_l_-Experiment 1) when adjusting for isolator effect. **Table S9.** Differentially abundant pathways in common for the comparisons between lines N and 6_1_ and between high and low carriers from line 6_l_ in Experiment 1.

## Data Availability

The sequencing data analysed during the current study are available in the NCBI Sequence Read Archive (SRA) database under the Bioproject accession number PRJNA649900. All data generated and analysed during this study are included in this published article and its additional files.
